# PcsB Expression Diversity Influences on *Streptococcus mitis* Phenotypes Associated With Host Persistence and Virulence

**DOI:** 10.3389/fmicb.2019.02567

**Published:** 2019-11-12

**Authors:** Erika N. Harth-Chu, Lívia A. Alves, Jéssica D. Theobaldo, Mariana F. Salomão, José F. Höfling, William F. King, Daniel J. Smith, Renata O. Mattos-Graner

**Affiliations:** ^1^Department of Oral Diagnosis, Piracicaba Dental School, UNICAMP, Piracicaba, Brazil; ^2^Department of Immunology and Infectious Disease, The Forsyth Institute, Cambridge, MA, United States

**Keywords:** *Streptococcus mitis*, PcsB, GbpB, biofilm, exopolysaccharides, complement immunity, virulence, microbial ecology

## Abstract

*S. mitis* is an abundant member of the commensal microbiota of the oral cavity and pharynx, which has the potential to promote systemic infections. By analyzing a collection of *S. mitis* strains isolated from the oral cavity at commensal states or from systemic infections (blood strains), we established that *S. mitis* ubiquitously express the surface immunodominant protein, PcsB (also called GbpB), required for binding to sucrose-derived exopolysaccharides (EPS). Immuno dot blot assays with anti-PcsB antibodies and RT-qPCR transcription analyses revealed strain-specific profiles of PcsB production associated with diversity in *pcsB* transcriptional activities. Additionally, blood strains showed significantly higher levels of PcsB expression compared to commensal isolates. Because *Streptococcus mutans* co-colonizes *S. mitis* dental biofilms, and secretes glucosyltransferases (GtfB/C/D) for the synthesis of highly insoluble EPS from sucrose, profiles of *S. mitis* binding to EPS, biofilm formation and evasion of the complement system were assessed in sucrose-containing BHI medium supplemented or not with filter-sterilized *S. mutans* culture supernatants. These analyses showed significant *S. mitis* binding to EPS and biofilm formation in the presence of *S. mutans* supernatants supplemented with sucrose, compared to BHI or BHI-sucrose medium. In addition, these phenotypes were abolished if strains were grown in culture supernatants of a *gtfBCD*-defective *S. mutans* mutant. Importantly, GtfB/C/D-associated phenotypes were enhanced in high PcsB-expressing strains, compared to low PcsB producers. Increased PcsB expression was further correlated with increased resistance to deposition of C3b/iC3b of the complement system after exposure to human serum, when strains were previously grown in the presence of *S. mutans* supernatants. Finally, analyses of PcsB polymorphisms and bioinformatic prediction of epitopes with significant binding to MHC class II alleles revealed that blood isolates harbor PcsB polymorphisms in its functionally conserved CHAP-domain, suggesting antigenic variation. These findings reveal important roles of PcsB in *S. mitis*-host interactions under commensal and pathogenic states, highlighting the need for studies to elucidate mechanisms regulating PcsB expression in this species.

## Introduction

*Streptococcus mitis* is abundant in multiple oropharyngeal sites, including mucosal and dental surfaces (Aas et al., 2005; Human Microbiome Project, 2012). Although classically recognized as a commensal organism, this species also emerges as an important opportunistic pathogen of systemic infections (Mitchell, 2011). The broad profile of *S. mitis* interactions with host sites compared to other streptococcal species might result from its high genetic and phenotypic diversity (Bek-Thomsen et al., 2008; Mitchell, 2011; Kilian et al., 2014). However, the molecular functions of *S. mitis* strains involved in host colonization as commensals and/or as opportunistic pathogens are poorly understood. Taxonomically grouped into the Mitis group along with *Streptococcus oralis* and *Streptococcus pneumoniae* (Facklam, 2002), *S. mitis* typically co-inhabits mucosal niches of streptococcal pathogens including *Streptococcus pyogenes* and *S. pneumoniae*, as well as other commensal streptococci, e.g., *Streptococcus salivarius* (Salivarius group) (Aas et al., 2005; Human Microbiome Project, 2012). *S. mitis* strains further participate in the colonization of dental surfaces (Diaz et al., 2006; Heller et al., 2016), interacting with commensal species of the Sanguinis group, and with species associated with caries pathogenesis (*Streptococcus mutans*).

Because the genomes of *S. mitis* strains harbor gene orthologs found in several streptococcal species co-habiting their major host niches (Johnston et al., 2010; Kilian et al., 2014; Lessa et al., 2018), it is likely that gene expression profiles of pioneer *S. mitis* strains influence immune responses to shared epitopes expressed by related species under commensal or pathogenic life-styles (Smith and Mattos-Graner, 2008; Nogueira et al., 2012; Lessa et al., 2018). One of these conserved antigens is PcsB (Protein required for cell wall separation of group B *Streptococcus,* PcsB), also known as GbpB (Glucan-binding protein B) in *S. mutans* (Mattos-Graner et al., 2001). In *S. pneumoniae* and *S. mutans*, PcsB/GbpB are secreted and surface-associated proteins involved in cell wall division (Ng et al., 2003; Duque et al., 2011; Sham et al., 2011) and/or surface interaction with exopolysaccharides (EPS) during biofilm formation (Mattos-Graner et al., 2001, 2006). Importantly, high titers of salivary IgA antibody to *S. mutans* GbpB or of serum IgG antibody to *S. pneumoniae* PcsB are naturally developed in young children and/or adults, who show limited carriage of these respective species, suggesting protective effects of anti-GbpB/PcsB antibodies (Nogueira et al., 2005; Giefing et al., 2008). The reasons for these individual robust immune responses to GbpB/PcsB remain to be elucidated. One hypothesis is that expression of GbpB/PcsB orthologs by pioneer strains of commensal streptococci, e.g., *S. mitis*, might prime immune responses to conserved epitopes capable of modulating persistence of more pathogenic lineages. However, there is no information about conservation of PcsB epitopes within *S. mitis* streptococci, nor about *S. mitis* profiles of PcsB expression associated with virulence and persistence traits. Therefore in this study, we assessed *pcsB* polymorphisms within conserved PcsB epitopes, and investigated whether diversity in PcsB expression within *S. mitis* strains isolated from different host sites could be associated with PcsB-mediated phenotypes.

## Materials and Methods

### Bacterial Strains, Plasmids, and Growth Conditions

A total of 20 *S. mitis* strains were included in this study (Table 1). Twelve of these strains were isolated from seven healthy infants who were 2–16 months of age, and who attended an oral health education and prevention program of the *Centro de Pesquisas e Atendimento Odontológico para Pacientes Especiais* (CEPAE) of the Piracicaba Dental School, State University of Campinas (UNICAMP), SP, Brazil. These isolates were obtained from the oral mucosal sites (gingival crevices, cheeks, palate and tongue dorsum) with sterile swabs, using a protocol previously approved by the Ethics Committee of FOP-UNICAMP (protocol 055/2010), as previously described (Palma et al., 2016). These isolates were identified as those of *S. mitis* species by using an identification system with specific primers (Garnier et al., 1997) and confirmed by sequencing of *16S rRNA* genes. Eight *S. mitis* strains with available genomes at the GenBank^1^, which were isolated from the oral cavity/dental biofilms (*n* = 3) or from the bloodstream of patients with clinical symptoms of bacteremia or septicemia (*n* = 5) were also analyzed. These strains were provided by Dr. Mogens Killian (Aarhus University, Denmark) and were deposited at CCUG as indicated in Table 1. Clinical backgrounds of patients harboring the blood strains SK579, SK569, and SK575 were previously described (Bochud et al., 1994). There is no published information about clinical conditions of subjects harboring strains SK616 and SK1073. The streptococcal strains were cultured in BHI (37°C) under aerobic (10% CO_2_ in air or aerobic shaking) or anaerobic (80% N_2_, 10% CO_2_, 10% H_2_) atmospheres. *Escherichia coli* strains TOP10 and BL21 were aerobically grown in Luria-Bertani medium supplemented with ampicillin (100 mg/ml) when required.

**Table 1:** Streptococcal strains used in this study.

Strain designation	Site of isolation, or relevant characteristic	Source and/or reference
***Streptococcus mitis***		
NCTC12261^a^^,^^*^	Oral cavity	Mogens Kilian^†^
35–15	Oral cavity, infant	This study
38–4	Oral cavity, infant	This study
38–5	Oral cavity, infant	This study
38–7	Oral cavity, infant	This study
38–10	Oral cavity, infant	This study
38–15	Oral cavity, infant	This study
39–5	Oral cavity, infant	This study
22–14	Oral cavity, infant	This study
22–15	Oral cavity, infant	This study
26–2	Oral cavity, infant	This study
28–3	Oral cavity, infant	This study
3B-12	Oral cavity, infant	This study
SK579^b^^,^^*^	Blood	Mogens Kilian^†^; Bochud et al. (1994)
SK616^c^^,^^*^	Blood	Mogens Kilian^†^
SK569^d^^,^^*^	Blood	Mogens Kilian^†^; Bochud et al. (1994)
SK575^e^^,^^*^	Blood	Mogens Kilian^†^; Bochud et al. (1994)
SK1073^f^^,^^*^	Blood	Mogens Kilian^†^
SK137^*^	Dental biofilm	Mogens Kilian^†^
SK138^*^	Dental biofilm	Mogens Kilian^†^
***Streptococcus mutans***		
UA159^*^	Oral cavity, child with active caries	ATCC; Ajdić et al. (2002)
MT8148	Oral isolate, child	Kasuhiko Nakano^‡^; Nakano et al., 2002
BC7s	Δ*gtfD*::*erm^r^* Δ*gtfBC*::*kan^r^* triple mutant obtained in MT8148	Kasuhiko Nakano^‡^; Ooshima et al. (2001)

a *SK142*.

b *Originally designated isolate n^o^. H8295 from P. Francioli (Lausanne, Schwitzerland); deposited by M. Kilian as CCUG 62641*.

c *Originally designated isolate n^o^. 6680/95 from Statens Serum Institut, Copenhagen, Denmark; deposited by M. Kilian as CCUG 62642*.

d *Originally designated isolate n^o^. H2112 from P. Francioli (Lausanne, Schwitzerland), deposited by M. Kilian as CCUG 62643*.

e *Originally designated isolate n^o^. H5422b from P. Francioli (Lausanne, Schwitzerland); deposited by M. Kilian as CCUG 62644*.

f *CCUG 47273; from Blood Dept, PHLS, Göteborg, Sweden*.

* *Genomes available at NCBI-GenBank (https://www.ncbi.nlm.nih.gov/genome/genomes/)*.

† *Provided by Dr. Mogens Kilian, Aarhus University, Denmark*.

‡ *Provided by Dr. Kazuhiko Nakano, Osaka University, Japan*.

### Analyses of PcsB Polymorphisms and Conserved Epitopes

Complete sequences of *pcsB* were obtained from all the studied strains. Briefly, genomic DNAs were purified using Master Pure DNA purification kit (Epicenter Technologies, Madison, WI, USA), and applied for amplification of the chromosomal region located 84 bp upstream to 169 bp downstream of *pcsB* encoding region, using primer sets designed using the genome of strain NCTC12261 as reference (Table 2). Amplicons were sequenced using a 3,500 Genetic Analyzer 8 capillary sequencer (Applied Biosystems HITACHI) and sequences edited using BioEdit 7.2.5^2^. Multiple sequence alignments were performed using ClustalW^3^ and/or BoxShade v 3.21 tools^4^. A similarity cladogram of *S. mitis* PcsB and orthologous proteins of oropharyngeal streptococci were obtained using the Phylogeny.fr platform (MABL; http://www.phylogeny.fr/) (Dereeper et al., 2008, 2010). Orthologous protein of *Enterococcus faecium* DO (secreted antigen A; GenBank accession number YP_006377165.1) was used as outgroup in the phylogenetic comparisons. Potential peptides with binding affinity to human alleles of Major Histocompatibility Complex (MHC) class II molecules within PcsB sequences were identified using Tepitope/Proped bioinformatics tool^5^ (Singh and Raghva, 2001).

**Table 2:** Oligonucleotides and plasmids used in this study.

Primers or plasmid designation	Sequence (5′–3′)^a^ or relevant traits	Product size, position, or optimal melting temperature
***PcsB* sequencing**		
*pcs*BMIProm-For *pcs*BMIProm-Rev	GTGGTAGGTTTAACTGTGGA GATAGTGCTTGAACTTGTGC	1,010 bp, 216 bp upstream of to 794 bp downstream of the *pcsB* encoding region, 53°C
*pcs*BMIFor-all *pcs*BMIRev-all	AAAAATGTAACAAAGGCGTAA CAAAGGCAACTGTTTCACAA	1,500 bp, 84 bp upstream to 169 downstream of the *pcsB* encoding region, 55°C
*pcs*BMIFor-Int *pcs*BMIRev-Int	GCAAGTCAACAACAAACAGTAG CAAAGGCAACTGTTTCACAA	691 bp, 748 bp downstream of *pcsB* start codon to 169 bp downstream of stop codon, upstream of stop codon, 55°C
**qPCR**		
*16S*miRTFor *16S*miRTRev	ATGAGTTGCGAACGGGTGAG GCTATGTATCGTCGCCTTGGT	201 bp, 54°C
*pcs*BmiRTFor *pcs*BmiRTRev	AACAGCACAACAACAAGAAG GTTTGAGCACTACGAGCTT	208 bp, 54°C
***pcsB* cloning**		
*pcs*Bmi-F *pcs*Bmi-R	GGCCATGGAAACTACTGATGACAAAATTGCT GGCTCGAGGTTTGGATAGATATAT TGTTACAAAACC	1,167 bp, 68°C
**Plasmid for *pcsB* cloning**		
pET22b+	5,493 bp, Amp^r^, Novagen	–

a *Underlined sequences indicate restriction enzyme linkers*.

### Recombinant PcsB Protein and Monoclonal Antibodies

Recombinant His-tag *S. mitis* PcsB (rPcsB) was obtained by cloning the *pcsB* encoding region (amplified from strain NCTC12261 using specific primers; Table 2) into NcoI and XhoI cloning sites of plasmid pET22b + (Novagen) to yield PET-pcsB. PET-pcsB was transformed into *E. coli* BL21, and the recombinant protein isolated from 1 l cultures in LB medium supplemented with ampicillin (100 mg/ml) (A_550nm_ 0.5) after induction of rPcsB expression during 3–4 h with 1 mM isopropyl-β-D-thiogalactopyranoside (IPTG). Afterward, rPcsB was purified by affinity chromatography using the Ni-NTA Purification System (Thermo Fisher Scientific, U.S.A), as described elsewhere (Camargo et al., 2018). Samples were dialyzed in phosphate buffered saline (PBS) at 4°C, and stored at −20°C until use. Protein extracts were monitored in 10% SDS-PAGE gels stained with Coomassie blue. Monoclonal antibodies (MAb) against rPcsB were produced in mice using standard protocols (Rhea Biotech, SP, Brasil). Specificity of anti-PcsB MAbs were analyzed in ELISA and western blot assays with rPcsB and with cell extracts of *S. mitis* NCTC12261.

### Preparation of Protein Extracts of *S. mitis* Strains

Production of secreted and cell-associated PcsB was analyzed respectively in culture supernatants and whole cell extracts of cells grown in BHI under aerobic and anaerobic conditions until the mid-log phase of growth (A_550nm_ 0.3). Briefly, adjusted numbers of cells from 18 h cultures in BHI were transferred to 25 ml of fresh BHI medium and incubated (37°C) under aerobiosis (shaking at 160 rpm) or anaerobiosis (10% CO_2;_ 10% H_2_; 80% N_2_) until the A_550nm_ 0.3. The culture supernatants of these cultures were collected by centrifugation (twice at 6,000 × *g*; 4°C; 4 min), neutralized by addition of 1 M NaOH and 10 μM of phenylmethylsulfonyl fluoride (PMSF), and stored at −70°C until use. For preparation of whole cell extracts, the cells harvested from volumes of 25 ml of the same cultures were washed twice with saline solution, suspended in 2 ml of MilliQ water, and mechanically disrupted in a Bead Beater (Biospec Products) with 0.16 g of 0.1-mm zirconia beads (2 cycles of 45 s with 1 min rest on ice). Extracts were then centrifuged (12,000 × *g*; 4°C; 1 min) and the supernatants stored at −70°C until use. Culture supernatants were dialyzed (overnight at 4°C) against cold phosphate buffer (PB; 0.2 M; pH 6.5), and then against cold Tris-HCl (0.125 M; pH 6.8; diluted 1:100). Samples were then 100-fold concentrated by freeze-drying. The protein concentrations of samples were determined using a Bradford assay (Sigma) according to the manufacturer’s protocol.

### Imuno Dot Blot Assays

Amounts of PcsB in culture supernatants and cell extracts were quantified in immune dot blot assays, using MAbs anti-*S. mitis* rPcsB. Dot blotting of cell extracts or culture supernatants was performed as described (Mattos-Graner et al., 2001) with minor modifications. Briefly, nitrocellulose membranes (BioRad, CA, USA) were washed with phosphate buffer (PB) (0.2 M; pH 6.8) and applied to the dot blot apparatus (BioRad). Wells were then washed twice with 200 ul of PB, and 100 μl of culture supernatants or of protein extracts (equivalent to 10 μg of total protein) was applied per well. Samples of serially diluted rPcsB (1.95–250 ng) were also applied to each membrane for standard curves. After sample drainage, wells were washed twice with PB under vacuum, and the membranes removed and blocked with PBS supplemented with 5% skim milk (37°C under stirring, 60 min.). Afterward, the membranes were washed with PBS and incubated with anti-rPcsB MAbs (1:1,000) during 1.5 h at rt. After a new series of washes with PBS, the membranes were incubated with goat anti-mouse IgG antibody conjugated with horseradish peroxidase (1:10,000) (Thermo Scientific) (1.5 h at rt). As negative controls, membranes blotted with standard protein extracts obtained from a reference strain (NCTC12261) were probed only with the secondary antibody at the same conditions. PcsB probing was detected using the chemiluminescent SuperSignal West Dura system (Thermo Scientific, MA, USA), according to the manufacturer’s instructions, and converted by digital images using a GS-700 Imaging Densitometer. The intensities of PcsB signals for each well were then measured using the ImageJ 1.47 t software^6^ and converted to nanograms of PcsB based on the standard curves obtained in each blot. Relative levels of PcsB produced by each strain were obtained by summing of the respective measures of PcsB in culture supernatant and cell extract samples, which were expressed as arbitrary values. Three independent experiments were performed.

### RNA Isolation and Reverse Transcription qPCR

Amounts of *pcsB* transcripts were determined in *S. mitis* at mid-log growth phase (A_550nm_ 0.3), under aerobic or anaerobic conditions. Briefly, cells were harvested (6,000 × *g*; 4°C; 5 min.) from 25 ml of BHI cultures, resuspended in 1 ml of 0.9% saline, and frozen at −70°C. Afterward, these cells were mechanically disrupted in a Mini-BeadBeater (Biospec) with 0.16 g zirconia beads (0.1 mm diameter). RNA was isolated using a modified protocol from the RNeasy Mini kit (Qiagen) described elsewhere (Stipp et al., 2013). Briefly, disrupted cells were homogenized in RLT buffer (850 μl) and centrifuged (10,000 × *g*, 1 min, 4°C) for collection of the supernatants (700 μl), which were then mixed with ethanol (500 μl) and loaded onto columns. Further RNA purification steps were performed as recommended by the manufacturer. Afterward, samples were treated with 10 U Turbo DNase (Ambion) according to the manufacturer’s protocol, for removal of DNA. One microgram of RNA was then used for reverse transcription (RT) with random primers, using the SuperScript III system (Thermo Fisher Scientifc, USA), as described elsewhere (Stipp et al., 2008). Quantitative PCR was performed in a StepOne real-time PCR system (Thermo Fisher USA) in reaction samples (10 μl) containing 1 μl of cDNA samples, 1X Power SYBR green PCR master mix (Lifetech), and 10 μM of each primer for *16SrRNA* or for *pcsB* (Table 2). The cycling conditions included incubation at 95°C (10 min), followed by 40 cycles of 95°C (15 s), 54°C (15 s), and 72°C (30 s). Assays were performed in duplicate with RNA samples obtained from three independent experiments.

### Analysis of *S. mitis* Interactions With Sucrose-Derived Exopolysaccharides and Biofilm Formation

*S. mitis* interactions with sucrose-derived EPS were analyzed as previously described (Alves et al., 2016), in BHI with 1% sucrose supplemented or not with cell-free culture supernatants of *S. mutans* MT8148, as a source of glucosyltransferases (GtfB, GtfC, and GtfD). These three secreted enzymes are required for optimal synthesis of highly stable insoluble glucan EPS from sucrose by *S. mutans* (Ooshima et al., 2001; Bowen and Koo, 2011). As controls, culture supernatant of a triple Δ*gtfBCD* mutant obtained in MT8148 (designated BC7s) (Ooshima et al., 2001) was also used in these assays. Briefly, volumes of 5 ml of BHI or of filter-sterilized BHI culture supernatants of *S. mutans* strains (pH ≈ 6.6–6.8) were supplemented with 1% sucrose and inoculated with adjusted numbers of *S. mitis* tested strains. Samples were then incubated (37°C, 10% CO_2_ in air) for 18 h, and then the intensity of aggregation was scored visually from 0 to 3. To prepare the culture supernatants, the *S. mutans* strains (MT8148, BC7s, or UA159) were grown in 25 ml of BHI medium (37°C, 10% CO_2_ in air) until the mid-log phase of growth (A_550nm_ 0.3, pH ≈ 6.6–6.8). Afterward, these cultures were centrifuged (6,000 × *g*; 4°C; 10 min), and the obtained supernatants sterilized by filtration through membranes with pores 0.22 μm in diameter (Corning®). As controls, *S. mitis* cultures were also mixed with *S. mutans* culture supernatants not supplemented with sucrose, or with fresh BHI with 1% sucrose.

Biofilm formation was assessed in microtiter plates as previously described (Mattos-Graner et al., 2001), with modifications. Briefly, BHI or filter-sterilized BHI culture supernatants of *S. mutans* (MT8148 or BC7s) supplemented with 1% sucrose were inoculated with adjusted numbers of *S. mitis* cells, and transferred in four replicates (200 μl per well) to polystyrene 96-well plates (flat-bottom; Cralplast). After incubation (37°C, 10% CO_2_ in air) for 18 h, plates were washed with distilled water to remove loosely attached cells, and the biofilms were stained with crystal violet. Stain was then eluted from biofilms in ethanol (30 min. at room temperature), and the absorbances of the eluates (A_575nm_) were expressed as indirect measures of biofilm biomass. The planktonic growth (A_550nm_) was assessed in the same cultures used in the biofilm assays to monitor bacterial growth.

### Analysis of C3b Deposition on *S. mitis* Strains

Binding of C3b to *S. mitis* strains grown in BHI, BHI 1% sucrose, or in BHI *S. mutans* UA159 culture supernatant supplemented with 1% sucrose was assessed as previously described (Alves et al., 2016), with modifications. Serum samples applied in these assays were collected from one volunteer who showed standard serum levels of C3 and IgG immunoglobulins, and reference profiles of C3b-mediated opsonization (Alves et al., 2016, 2019), according to a standard protocol previously approved by the Ethical Committee of the Piracicaba Dental School, State University of Campinas, SP, Brazil (proc. n° 153/2014). Briefly, strains were grown until the A_550nm_ 0.3, and approximately 10^7^ CFU of cells were harvested by centrifugation (10,000 × *g*, 4°C), washed twice with PBS (pH 7.4), and incubated (37°C, 30 min) with 20% serum (in PBS). Cells were then washed twice with PBS-Tween 0.05% (PBST), and incubated (on ice, during 40 min.) with fluorescein isothiocyanate (FITC)-conjugated polyclonal goat anti-human C3 IgG antibody (ICN, CA, U.S.A) (1:300 in PBST). Afterward, cells were washed twice with PBST and fixed in 3% paraformaldehyde (in PBS) for analysis on a FACSCalibur flow cytometer (BD Biosciences). Levels of surface-bound C3b on C3b-positive cells were expressed as the geometric mean of fluorescence intensity (MFI), using forward and side scatter parameters to gate at least 25,000 bacteria. Control samples included bacteria treated only with PBS. Heat-inactivated sera (56°C for 20 min) were also used as negative controls in preliminary experiments.

### Statistical Analysis

Phenotypic comparisons were performed using Kruskal-Wallis with *post hoc* Dunn’s multiple comparisons or Mann-Whitney tests. Spearman’s rank correlation was applied to analyze associations between relative amounts of PcsB produced and transcript levels of *pcsB*. Differences were considered significant when values of *p* ≤0.05 were obtained.

## Results

### Gene Structure and Conserved Immunogenic Epitopes of PcsB in *S. mitis* and Other Oropharyngeal Streptococci

**Figure 1:**
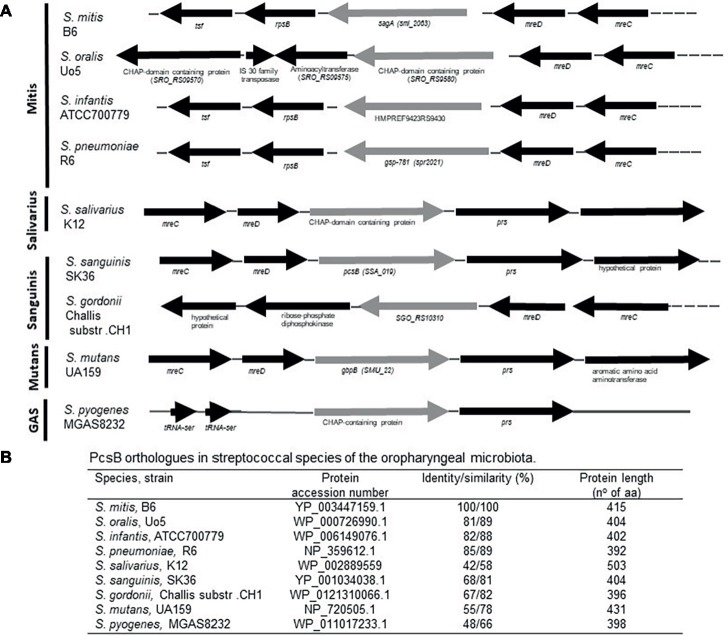
Comparative analyses of *pcsB* gene structure and homology in streptococcal species of the oral cavity and pharynx. **(A)** Schematic representation of the *pcsB* chromosomal *loci* in representative strains of eight species of the Mitis, Salivarius, Sanguinis, Mutans groups and of *S. pyogenes* (strain MGAS8232). Arrows represent direction of gene transcription; genes encoding the PcsB orthologs are represented in gray. Gene organization and designation were obtained from the GenBank database (http://www.ncbi.nlm.nih.gov/). **(B)** Results of BLASTp analyses using *S. mitis* PcsB sequence.

**Figure 2:**
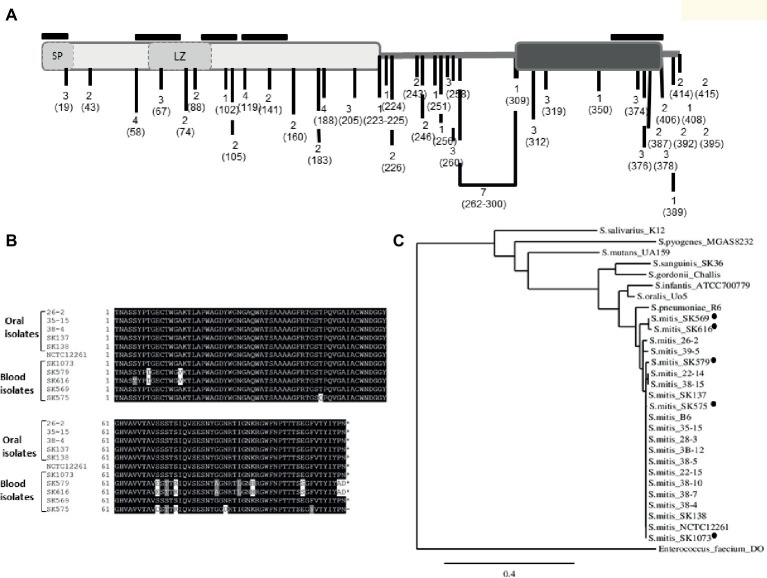
Polymorphisms of PcsB in *S. mitis* strains. **(A)** Schematic representation of *S. mitis* PcsB precursor protein with conserved functional domains. The PcsB N-terminal and C-terminal parts, respectively represented by light and dark gray rectangles, are linked by an alanine-rich variable region, which is represented by the bold line. In the N-terminal part, the signal peptide (SP) and the leucine zipper (LZ) motifs are represented by dashed rectangles. The frequencies of protein polymorphisms identified within 20 strains analyzed are indicated with numbers; the position of the amino acid changes is also indicated within parenthesis. The positions of five T cell epitope peptides are indicated with black lines, above the protein scheme. **(B)** BoxShade alignment of PcsB CHAP-domain sequences determined in five blood strains and in five representative oral strains. **(C)** Similarity cladogram of *S. mitis* PcsB and streptococcal orthologous proteins obtained using the Phylogeny.fr platform (http://www.phylogeny.fr/). *S. mitis* blood strains isolated from systemic infections are indicated with black circles. Orthologous protein of *Enterococcus faecium* DO (annotated as SagA) was used as outgroup.

**Figure 3:**
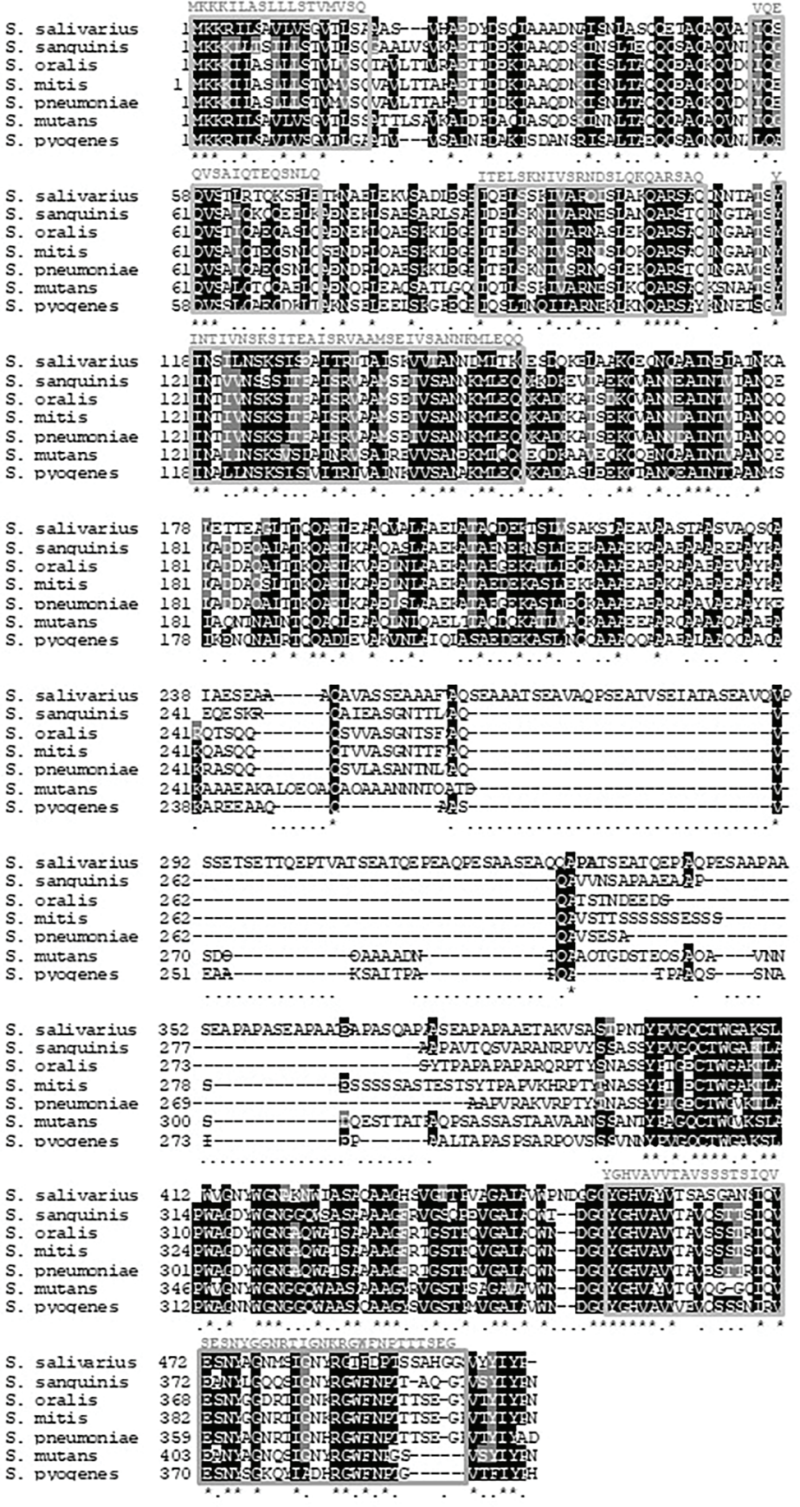
Localization of peptides with significant binding potential to MHC class II human alleles in PcsB orthologs. Sequences of PcsB orthologs identified in streptococcal species of the oral cavity and pharynx were aligned using BoxShade v 3.21 tools (https://embnet.vital-it.ch/software/BOX_form.html); asterisks indicate conserved residues; dots indicate non conserved residues. Five PcsB epitopes located at PcsB functional domains are indicated within boxes; predicted *S. mitis* PcsB epitope sequences are indicated above the respective boxes.

BlastP analysis of *S. mitis* PcsB was initially used to investigate conservation of PcsB orthologs within streptococcal species of the oropharynx. The genomic structures of *pcsB loci* were then analyzed. PcsB orthologs identified in the analyzed genomes were annotated as SagA (secreted antigen), GSP-781 (general stress protein-781), CHAP-domain containing protein, or GbpB (Glucan-binding protein B). The degree of protein similarity and gene structure of *pcsB* loci were compatible with phylogenetic relationships of the species analyzed (Figure 1). Except for the *S. oralis* strain Uo5, gene structure or *pcsB* loci were highly conserved within the Mitis species (Figure 1A). In these species, genes located downstream to *pcsB* encode putative 30S ribosomal proteins (*rpsB*). Differently in *S. oralis* strain Uo5, the *pcsB* downstream genes included a gene encoding an additional “CHAP-domain containing protein,” which was spanned by genes encoding transposases. Upstream to *pcsB* genes, two genes encoding putative cell-shape determining proteins (*mreC/D*) were highly conserved in all streptococcal species analyzed, except for *S. pyogenes*. In Salivarius, Sanguinis, and Mutans group species, as well as in *S. pyogenes* strain MGAS8232, genes encoding ribose-phosphate diphosphokinase protein (*prs*) were located downstream to *pcsB* genes. We could not identify a PcsB ortholog in the *S. pyogenes* strain M1 GAS SF370. *S. mitis* PcsB shows high amino acid sequence similarity with most of the oral species (Figure 1B). The genomes of *S. salivarius* SK12 and *S. pyogenes* strain MGAS8232 include proteins with the lowest percentages of similarity with *S. mitis* PcsB (Figure 1B). Apart from differences in protein identity/similarity, all the PcsB orthologs found in the streptococcal species show a typical domain structure. These include an N-terminal signal peptide for protein secretion (amino acids 1–27 in *S. mitis*), by a leucine zipper domain (amino acids 65–93 in *S. mitis*), a variable alanine-rich linker region, and a C-terminal CHAP domain (Cys, His-dependent amidohydrolase/peptidase) with two conserved residues (Cys315 and His366) involved in peptidoglycan hydrolytic activity (Figure 2A). Because PcsBs were found to be immunodominant antigens of *S. mutans* and *S. pneumoniae* (Smith et al., 2003; Nogueira et al., 2005; Giefing-Kröll et al., 2011), we further investigated potential PcsB T epitopes with binding affinity to human MHC class II alleles (Tepitope/Proped tool). These bioinformatic analyses revealed five T epitopes localized in the most conserved part of PcsB proteins (Figures 2A, 3). Four of them were located in the N-terminal part of PcsB, and included sequences within or spanning the signal peptide and the leucine zipper domains (Figures 2A, 3). An additional C-terminal epitope was located in the CHAP domain. Therefore, PcsB T epitopes are located in functional regions, which are conserved within different streptococcal species of the oral cavity and pharynx.

### The Existence of Polymorphisms in *S. mitis* PcsB Proteins Associated With Functional Domains and Potential Immunogenic Epitopes

Comparisons of *pcsB* of the 20 studied strains (using strain NCTC12261 as reference) revealed several polymorphisms associated with amino acid changes within conserved functional domains, as well as insertion and deletions at the variable linker region. Figure 2A illustrates the frequency of amino acid changes within strains and the position of these protein polymorphisms, in relation to functional domains and to predicted T epitopes. A significant proportion of amino acid changes were within the five putative MHC class II peptides, and several of these mutations (*n* = 16) were within the C-terminal extremity of the CHAP-domain (position 305–385). Interestingly, most of the mutations on MHC class II peptides were present in three blood isolates (SK575, SK579, and SK616). The blood strain SK569 also accounted for a significant number of amino acid changes (*n* = 14), but most of them were located within the four N-terminal epitopes. The strains isolated from the oral cavity of infants showed a reduced number of polymorphisms associated with T epitopes (*n* = 2), and most of the polymorphisms found in these strains were not within functional domains. Figure 2B shows alignment of the N-terminal PcsB sequences containing the entire CHAP-domain (111 amino acids in length) of five blood strains as well as five representative oral strains. Phylogenetic analysis of PcsB sequences (Figure 2C) further shows that apart from polymorphisms identified within PcsB expressed by *S. mitis* strains, these proteins are more closely related compared to PcsB orthologs expressed by other species of oropharyngeal streptococci.

### Diversity in Production of PcsB in *S. mitis* Strains Associated With Transcriptional Activities of *pcsB*

**Figure 4:**
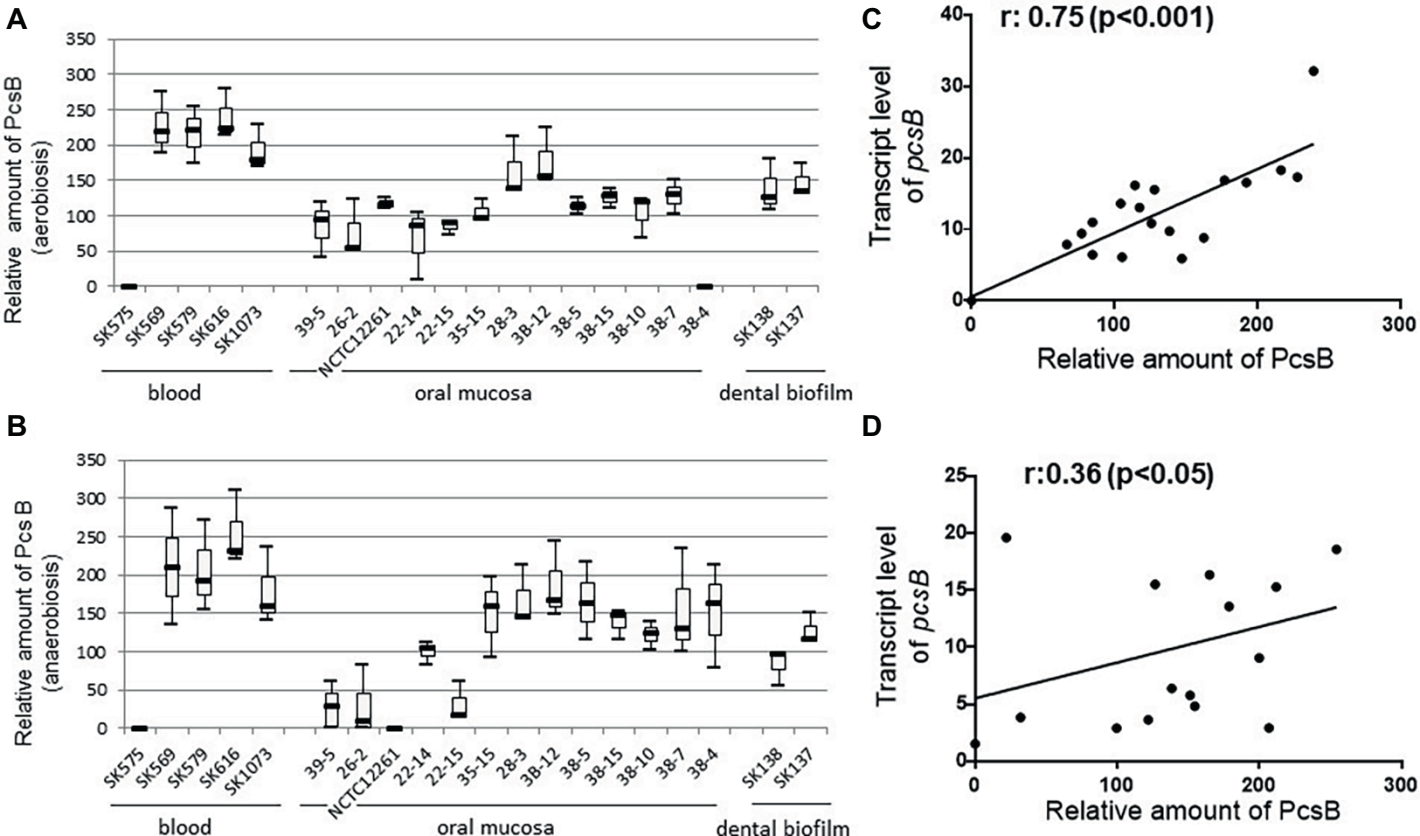
PcsB expression by *S. mitis* strains isolated from different host sites. Strains grown under aerobic **(A)** or anaerobic **(B)** conditions until the mid-log growth phase for collection of bacterial cells and culture supernatants, which were analyzed in immuno dot blot assays with MAbs specific to PcsB. Relative amounts of PcsB produced by each strain were measured by densitometry of the dot blots, under a linear range of PcsB detection. Horizontal lines within box plots indicate mean levels of three independent experiments; bars indicate standard deviations. Strain designations and sites of host isolation are indicated on the X-axis. **(C,D)** Spearman’s rank correlation analyses between relative amounts of PcsB produced and *pcsB* transcript levels (determined by RT-qPCR) in strains grown respectively at aerobic and anaerobic conditions.

Profiles of PcsB production and protein localization were initially investigated in the studied *S. mitis* strains using protein cell extracts and samples of culture supernatants at mid-log phase of growth. Measures of secreted and cell-associated PcsB were assessed at different atmospheric conditions (aerobiosis and anaerobiosis), because *S. mitis* colonizes oral niches with varying oxygen tensions. Immuno dot blot analyses of secreted (culture supernatants) and cell extracts revealed significant diversity in total amounts of PcsB produced between strains. Diversity in total amounts of PcsB produced (secreted plus cell-associated PcsB) under aerobic conditions (Figure 4A) was significantly associated with diversity in PcsB production under anaerobiosis (Figure 4B) (Spearmans’s correlation analysis: *r*: 0.80; *p* < 0.05). In both atmospheric conditions, most of the PcsB was present in the culture supernatants (means: 84.57 ± 43.58 and 67.81 ± 40.32 in aerobiosis and anaerobiosis, respectively) compared to the cell fractions (means: 2.37 ± 1.95 and 0.68 ± 1.52 in aerobiosis and anaerobiosis, respectively). To further investigate mechanisms underlying diversity in PcsB production, we analyzed *pcsB* transcriptional activities by RT-qPCR, using RNA samples obtained from cells harvested from the same cultures assayed for protein production. As shown in Figures 4C,D, there was significant positive association between amounts of PcsB production and transcript levels of *pcsB* in both atmospheric conditions, implying that diversity in PcsB production between strains was promoted by differences in transcriptional activities of the respective *pcsB* genes.

### *S. mitis* Strains Involved in Systemic Infections Show Increased Expression of PcsB and PcsB-Mediated Phenotypes

**Figure 5:**
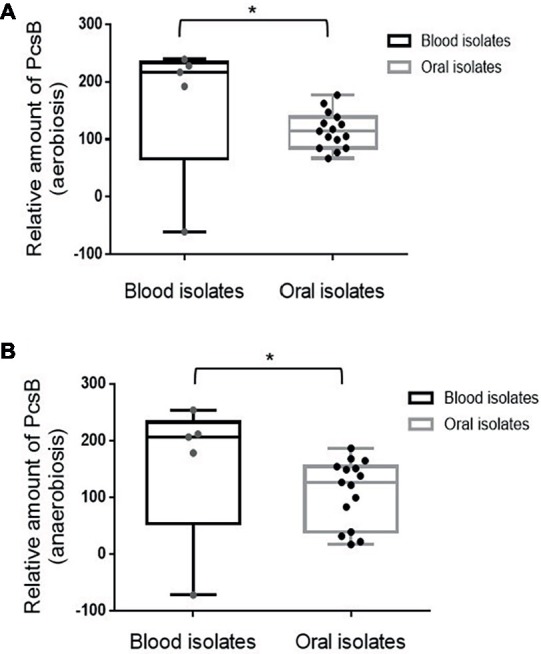
Box plot comparisons of PcsB production between *S. mitis* strains isolated from the bloodstream or from oral sites. Amounts of PcsB produced were determined using immuno dot blot assays in strains grown under aerobic **(A)** and anaerobic **(B)** conditions. Mean levels of PcsB are represented by horizontal lines within boxes. Bars represent standard deviations. Asterisks indicate statistically significant differences between groups in Mann-Whitney U-test (*p* < 0.05).

**Figure 6:**
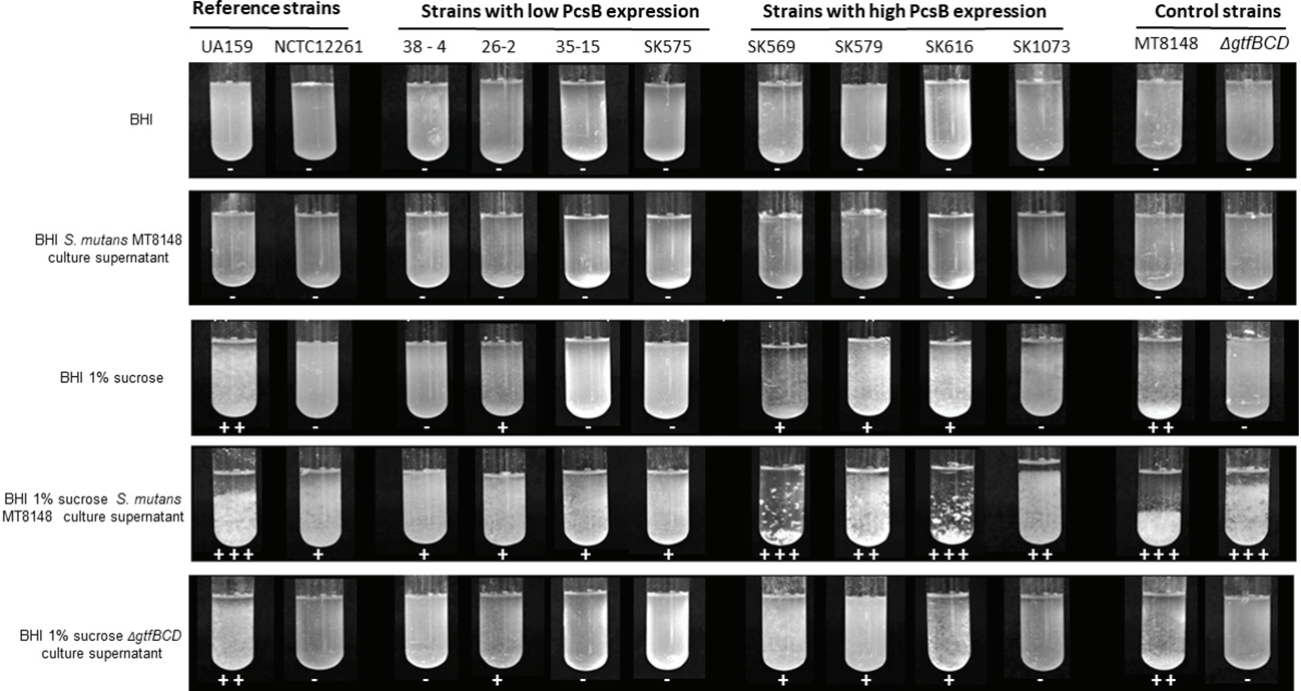
Analysis of bacterial binding to EPS. *S. mitis* strains with the highest (*n* = 4) and lowest (*n* = 4) levels of PcsB produced were grown in BHI or BHI with 1% sucrose supplemented or not with filter-sterilized culture supernatants of *S. mutans* strains (MT8148 or Δ*gtfBCD* isogenic mutant). Bacterial aggregation mediated by EPS synthesized from sucrose was then visually examined and the intensities of aggregation scored from 0 (−) to 3 (+++), as indicated below the respective cultures. The reference strains *S. mutans* UA159 and *S. mitis* NCTC12261 were also analyzed.

In *S. mutans*, up-regulation of genes involved in binding to sucrose-derived EPS is associated with systemic virulence (Alves et al., 2016). Thus, we compared profiles of PcsB production between *S. mitis* strains associated with systemic infections (blood strains) and strains isolated from oral sites at commensal states. As shown in Figure 5, blood strains produce increased amounts of PcsB, compared to oral strains, either under aerobic or anaerobic conditions. To further address if PcsB expression influences the strain capacity to bind sucrose-derived EPS, we selected strains with the highest (*n* = 4; strains SK569, SK579, SK616, SK1073) and lowest (*n* = 4; strains SK575, 38–4, 35–15, 26–2) levels of PcsB (produced under aerobic conditions) to compare bacterial aggregation mediated by sucrose-derived EPS. Of note, different from *S. mutans*, *S. mitis* does not secrete glucosyltransferase enzymes (Gtfs) required for the synthesis of insoluble glucan EPS from sucrose (Xu et al., 2018). Therefore, to supply *S. mitis* cultures with sucrose-derived EPS, *S. mitis* aggregation was assessed in filter-sterilized BHI culture supernatants of *S. mutans* MT8148 (which contain secreted Gtfs: GtfB, GtfC and GtfD) supplemented with sucrose. As controls of Gtf activities, we also supplemented *S. mitis* cultures with the culture supernatants of the *S. mutans gtfBCD* triple mutant obtained in MT8148. As shown in Figure 6, *S. mutans* reference strain UA159 and *S. mitis* strains show irrelevant aggregation in BHI without sucrose or in *S. mutans* supernatants in BHI without sucrose, the unique substrate of Gtfs. On the other hand, BHI supplementation with 1% sucrose promoted aggregation of *S. mutans* UA159, but this phenotype was less evident in most of the *S. mitis* strains. On the other hand, growth in BHI 1% sucrose medium supplemented with culture supernatants of *S. mutans* strain MT8148 clearly increased aggregation of *S. mitis* strains expressing high levels of PcsB, a phenotype not observed in low PcsB-expressing strains (Figure 6). Importantly, the enhanced aggregation phenotypes promoted by the supernatants of *S. mutans* MT8148 were abolished, when *S. mitis* strains were grown in BHI 1% sucrose medium with culture supernatants of the *gtfBCD*-defective strain (BC7s). Of note, aggregation of the *gtfBCD*-defective *S. mutans* was restored to parent levels, when this defective mutant was grown in BHI-sucrose medium supplemented with MT8148 culture supernatant.

**Figure 7:**
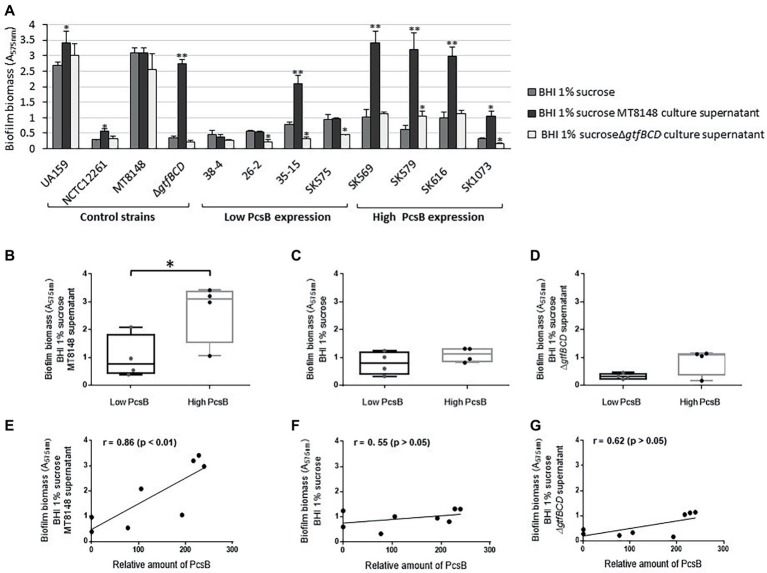
Comparisons of biofilm formation by *S. mitis* strains differing in PcsB expression levels. **(A)** Biofilm biomass was assessed in biofilms formed in microtiter plates by *S. mitis* strains grown in BHI 1% sucrose or in BHI 1% sucrose supplemented with culture supernatants of *S. mutans* MT8148 or Δ*gtfBCD* mutant strain. Columns represent means of four replicates of one representative experiment. Asterisks above columns indicate statistically significant differences in relation to the biofilms formed by the same strain in BHI 1% sucrose (Kruskal-Wallis with *post hoc* Dunn’s multiple comparisons: ^*^p < 0.05; ^**^p < 0.01). **(B–D)** Box plot comparisons of biofilm biomasses between strains expressing low (*n* = 4) versus high (*n* = 4) levels of PcsB, in BHI-sucrose MT8148 culture supernatant **(B)**, BHI-sucrose **(C)** or BHI-sucrose Δ*gtfBCD* mutant culture supernatant **(D)**. Asterisk indicates significant differences between groups (Mann-Whitney; ^*^p < 0.05). **(E–G)** Spearman’s correlation analysis between total levels of PcsB production and biomasses of biofilms formed in either BHI-sucrose MT8148 culture supernatant **(E)**, BHI-sucrose **(F)**, or BHI-sucrose Δ*gtfBCD* mutant culture supernatant **(G)**.

Because expression of *gbpB* is required for biofilm formation mediated by sucrose-derived EPS in *S. mutans* (Duque et al., 2011), and GbpB expression influences strain-specific capacities to form biofilms (Mattos-Graner et al., 2001), we compared the amounts of biofilms formed by *S. mitis* strains under the same culture conditions used to investigate EPS binding. As shown in Figure 7A, significant increases in biofilm biomass were observed in all high PcsB-expressing *S. mitis* strains grown in BHI-sucrose supplemented with *S. mutans* MT8148 culture supernatants, compared to biofilms formed in either BHI-sucrose or BHI-sucrose supplemented with Δ*gtfBCD* mutant culture supernatant. The *S. mutans gtfBCD*-defective mutant further showed a high increase in biofilm biomass when grown in BHI-sucrose supplemented with culture supernatants of parent strain MT8148. Comparisons of mean biofilm biomasses between *S. mitis* strains with low *versus* high PcsB expression further showed increased capacities of high PcsB producers to form biofilms in the presence of BHI-sucrose with MT8148 culture supernatant (Figure 7B). On the other hand, no significant differences between groups were detected when strains were grown in BHI-sucrose (Figure 7C) or BHI-sucrose with culture supernatants of the Δ*gtfBCD* mutant strain (Figure 7D). Consistently, correlation analysis revealed that PcsB expression levels were significantly associated with the biomasses of biofilms formed in BHI-sucrose supplemented with MT8148 culture supernatant (Figure 7E), but not with the biomasses of biofilms formed in BHI-sucrose medium (Figure 7F) or in BHI-sucrose with culture supernatants of the Δ*gtfBCD* mutant (Figure 7G). Growth of *S. mitis* strains in BHI-sucrose medium supplemented with culture supernatants of *S. mutans* UA159 instead of MT8148 resulted in similar EPS-binding and biofilm phenotypes (data not shown).

### *S. mitis* Strains Expressing Increased Levels of PcsB Show Resistance to Complement Deposition

**Figure 8:**
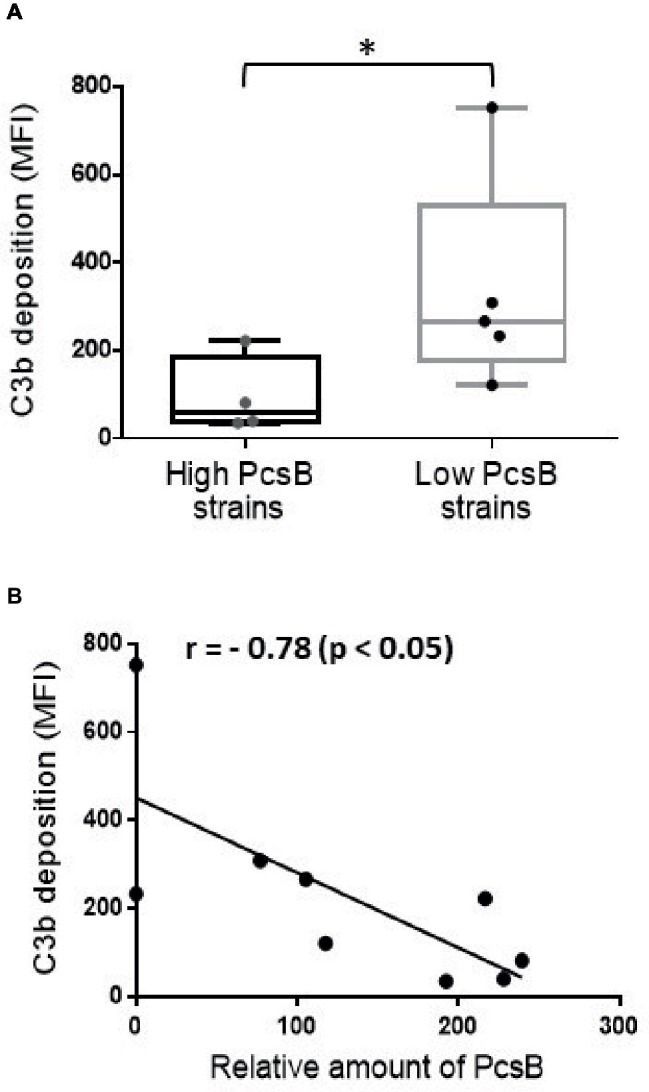
Association between PcsB expression and resistance to C3b deposition in *S. mitis* strains. Strains grown in *S. mutans* UA159 BHI culture supernatants supplemented with 1% sucrose (until A_757nm_ 0.3) were treated with 20% human serum, and surface-bound C3b probed with FITC-conjugated anti-human C3 antibody for quantification by flow cytometry; levels of surface-bound C3b were expressed as geometric means of fluorescent intensities (MFI). Asterisks indicate statistically significant differences between groups in Mann-Whitney U-test (*p* < 0.05). **(A)** Box plot comparisons of C3b deposition between strains with high (*n* = 4) and low levels (*n* = 5) of PcsB production. **(B)** Spearman’s correlation analysis between C3b deposition and relative amounts of PcsB produced.

*S. mutans* binding to sucrose-derived EPS promotes the formation of a capsule-like structure for evasion to complement immunity (Alves et al., 2016). We thus investigated whether *S. mitis* strains expressing high PcsB levels (SK569, SK579, SK616, SK1073) have resistance to complement deposition compared to low PcsB-producing strains (SK575, 38–4, 35–15, 26–2). These comparisons were performed with strains grown either in BHI, BHI 1% sucrose, or in BHI 1% sucrose supplemented with *S. mutans* UA159 culture supernatants. In all the tested conditions, high PcsB-expressing strains showed significantly lower levels of C3b binding compared to low PcsB producers (Mann-Whitney, *p* < 0.05; data not shown). The lowest levels of C3b deposition were observed when strains were grown in BHI 1% sucrose culture supernatants of *S. mutans* UA159. Figure 8A shows group comparisons of C3b binding at this growth condition. Correlation analysis between levels of C3b deposition on strains grown in BHI-sucrose medium with UA159 culture supernatant and levels of PcsB production further indicates influence of PcsB expression in complement evasion (Figure 8B). Therefore, increased levels of PcsB promote resistance to complement deposition in *S. mitis* strains at conditions that favor the synthesis of sucrose-derived EPS.

## Discussion

*S. mitis* is an abundant member of the oral microbiota of humans from early life to adulthood (Smith et al., 1993; Kononen et al., 2002; Aas et al., 2005; Bek-Thomsen et al., 2008; Human Microbiome Project, 2012), which implies commensal interactions with host immune functions. *S. mitis* species shows significant genomic and phenotypic diversity, and the genomes of strains isolated from opportunistic systemic infections, and at commensal states, include virulence gene orthologs typical of the pathogenic species *S. pneumoniae*, a close *S. mitis* relative (Whatmore et al., 2000; Kilian et al., 2008, 2014; Donati et al., 2010; Johnston et al., 2010). Evolutionary genomic comparisons of *S. mitis* and *S. pneumoniae* strains suggest that commensal behavior of *S. mitis* strains might be associated not simply with the absence of virulence-associated gene clusters, but rather with different profiles of expression of virulence functions (Donati et al., 2010; Kilian et al., 2014; Skov Sorensen et al., 2016). In this study, we show structural and expression diversity of PcsB in *S. mitis*, an immunodominant surface antigen conserved in streptococci and involved in phenotypes associated with bacterial persistence and virulence, including binding to sucrose-derived EPS, biofilm formation, and resistance to complement immunity. We further report polymorphisms in potential epitopes of MHC class II located at conserved PcsB functional domains, indicating antigenic variation. Finally, we established that most strains isolated from systemic infections not only have increased PcsB expression, but virulence phenotypes associated with PcsB molecular functions, when compared to commensal isolates.

PcsB/GbpB proteins are essential for bacterial viability in *S. pneumoniae* and *S. mutans*, likely due to their mureinolytic functions as part of the cell wall divisome (Mattos-Graner et al., 2006; Duque et al., 2011; Sham et al., 2011; Bartual et al., 2014). At least for *S. mutans*, this surface antigen is further involved in stable bacterial interaction with sucrose-derived glucan EPS (Mattos-Graner et al., 2001; Duque et al., 2011; Stipp et al., 2013), which are major components of the extracellular matrix of cariogenic dental biofilms (Bowen et al., 2018). In the present study, we show that diversity in PcsB production in *S. mitis* is associated with differences in transcriptional activities of *pcsB* genes. Different atmospheric conditions of growth (aerobiosis *versus* anaerobiosis) yield similar profiles of *pcsB* transcription and protein production, further indicating significant influence of the strain background on PcsB expression. These findings highlight the need for studies addressing mechanisms involved in *pcsB* transcriptional regulation in *S. mitis*.

In several streptococci of the oral cavity and pharynx, including *S. mutans, S. sanguinis,* and *S. pneumoniae*, *gbpB/pcsB* are directly regulated by the two-component system (TCS) VicRK (Ng et al., 2005; Senadheera et al., 2005; Stipp et al., 2013; Moraes et al., 2014). In these streptococci, VicRK typically regulates genes involved in virulence-associated functions, e.g., complement evasion, and/or biofilm formation (Ng et al., 2005; Senadheera et al., 2005; Duque et al., 2011; Alves et al., 2017). In *S. mutans*, several genes of the VicRK regulon, including *gtfB*, *gtfC,* and *gbpB*, are also co-regulated by CovR (Biswas and Biswas, 2006; Stipp et al., 2013), another regulator of virulence. CovR was characterized in more detail in *S. pyogenes*, as part of the TCS CovRS (Federle and Scott, 2002; Gryllos et al., 2007; Horstmann et al., 2017), but in *S. mutans* and in other oral species, CovR is an orphan regulator (Mattos-Graner and Duncan, 2017). In *S. pyogenes*, natural mutations affecting CovR activities were associated with increased expression of virulence-associated functions (Engleberg et al., 2001; Sumby et al., 2006). Orthologs of the TCS VicRK and CovR were found in the genome of *S. mitis* strain B6 (Mattos-Graner and Duncan, 2017). Thus, analysis of these transcriptional regulators in *S. mitis* strains might help to understand diversity in *pcsB* transcription in this species, as well as strain-specific virulence phenotypes.

GtfB, GtfC, and GtfD enzymes secreted by *S. mutans* account for biofilm enrichment through the synthesis of highly stable EPS (mostly insoluble glucan rich in α-1,3 glycosidic linkages) (Ooshima et al., 2001; Banas and Vickerman, 2003; Bowen and Koo, 2011). These enzymes retain activity once bound to the surface of bacterial species that typically co-habit dental biofilms (Bowen and Koo, 2011). Different from *S. mutans* and other streptococcal species of dental biofilms, the genomes of *S. mitis* strains do not harbor genes encoding Gtfs (Xu et al., 2018). This is consistent with the reduced *S. mitis* aggregation observed in BHI supplemented with sucrose, a phenotype also reported in *S. mutans gtfBCD* isogenic mutants (Alves et al., 2016). Because *S. mitis* strains are frequently detected during initial phases of biofilm formation (Diaz et al., 2006; Heller et al., 2016), as well as in cariogenic biofilms, which frequently harbor *S. mutans* (Nyvad and Kilian, 1990; Aas et al., 2008), we hypothesized that *S. mitis* expressing PcsB could interact with glucan EPS produced by GtfB/C/D secreted by *S. mutans*. *S. mitis* aggregation phenotypes were observed in the presence of *S. mutans* MT8148-secreted enzymes in BHI-sucrose medium (culture supernatants), but not in the presence of culture supernatants of the *ΔgtfBCD* isogenic *S. mutans* strain revealed that *S. mitis* not only interacts with EPS produced by *S. mutans*-secreted Gtfs, but that strains expressing increased levels of PcsB have enhanced capacity to bind these EPS and to form biofilms. *S. mitis* binding to EPS synthesized in the presence of *S. mutans* Gtfs was further associated with reduced levels of C3b deposition.

We have recently reported that *S. mitis* strains isolated from systemic infections have increased resistance to complement deposition, compared to oral strains (Alves et al., 2019), but differences in complement resistance could not be associated with the presence of the *cps* operon for the synthesis of capsule, as most of the strains harbored *cps* orthologs (Kilian et al., 2014; Skov Sorensen et al., 2016). Capsule expression is a major function of *S. pneumoniae* involved in complement evasion (Hyams et al., 2011), but differences in capsule composition and structure affect their protective functions (Whatmore et al., 2000; Hyams et al., 2013; Rukke et al., 2014; Lessa et al., 2018). In addition, *S. mitis* strains may co-express conserved or strain-specific gene orthologs potentially involved in complement evasion (e.g., *lytA*, *pepO*, *eno*, *gapdh*) (Agarwal et al., 2013; Lessa et al., 2018; Pimenta et al., 2018). Although capsule diversity, as well as expression of potential complement evasion genes remain to be investigated in the studied *S. mitis* strains, the present findings reveal an additional mechanism of *S. mitis* complement evasion. The findings that increased expression of PcsB by *S. mitis* promotes biofilm formation and complement evasion in a fashion dependent on interaction with *S. mutans*-derived components (i.e., EPS produced by *S. mutans* secreted enzymes) further exemplify how inter-species interactions could promote virulence phenotypes in commensal streptococci with incomplete panels of virulence-associated genes.

The role of PcsB in microbial interactions with extracellular components of biofilms implies that immune responses to PcsB could affect ecology of multiple species expressing PcsB orthologs. T epitope/ProPed bioinformatic analysis of GbpB/PcsB revealed five potential epitopes (Figure 2A) within PcsB functional domains conserved within several streptococcal species of the oral cavity and pharynx (Figure 3). The significant number of epitopes in the N-terminal part of PcsB is further consistent with prominent salivary IgA reactions with N-terminal linear peptides derived from *S. mutans* GbpB (Nogueira et al., 2008; Smith and Mattos-Graner, 2008). Cross-reactions of *S. mutans* and *S. mitis* antigens with salivary IgA were suggested in previous studies (Cole et al., 1999; Nogueira et al., 2012), although identities of the cross-reactive antigens remained unknown. Interestingly, *S. mitis* seems to also induce cross-reactive T CD4+ cell effector responses to *S. pneumoniae* (Engen et al., 2014). Our present report further indicates that pioneer colonization by *S. mitis* strains may prime cross-reactive adaptive responses to PcsB expressed by later colonizers. All the 12 studied strains isolated from the oral mucosa of young infants produced detectable levels of PcsB, thus would be capable of stimulating PcsB reactive IgA responses. Because several commensal species including *S. mitis* produce IgA1 proteases, which likely accounts for evasion to IgA-S effector functions (Kilian et al., 1995, 2008), it could be speculated that IgA antibody responses induced by commensal streptococci against PcsB would have a more prominent impact on the establishment of species that do not express these proteases, as is the case of *S. mutans*. In prospective studies of infants, natural salivary IgA antibody responses to *S. mutans* GbpB were evident during the first year of life in most children who were not colonized by this species a year later, whereas early colonized infants showed weak IgA responses to GbpB (Nogueira et al., 2005). Of note, *S. mutans* is more typically detected in the oral cavity by 19–30 months of age when most primary teeth erupt, the major niche of this species (Caufield et al., 1993; Smith et al., 1998), whereas *S. mitis* is detected shortly during the initial months of life (Smith et al., 1993). Thus, natural immune responses to GbpB/PcsB appear to affect initial colonization by *S. mutans* (Nogueira et al., 2005). In addition, experimental immunization of rats with GbpB significantly induces antibody responses to the same epitopes recognized by salivary antibodies of children (Nogueira et al., 2008), and protects animals from *S. mutans*-induced caries development (Smith et al., 2003). The present findings on *S. mitis* PscB raise further interest in defining the effects of immune responses to PcsB proteins in oral ecology and host-microbiota homeostasis.

## Data Availability Statement

The raw data supporting the conclusions of this manuscript will be made available by the authors, without undue reservation, to any qualified researcher.

## Ethics Statement

This study was carried out in accordance with the recommendations of the Ethical Committee of the Piracicaba Dental School, State University of Campinas, SP, Brazil. All subjects gave written informed consent in accordance with the Declaration of Helsinki. The protocol was approved by the Ethical Committee of the Piracicaba Dental School, State University of Campinas, SP, Brazil (proc. no. 055/2010 and 153/2014).

## Author Contributions

RM-G, DS, EH-C, LA, and WK conceived and designed the experiments. RM-G, EH-C, DS, and WK conceived and performed bioinformatic analyses. EH-C, JT, and MS isolated the *S. mitis* strains from infants, and performed phenotypic and genotypic analyses. EH-C and LA performed protein and transcriptional analyses. LA performed biofilm and complement deposition assays. RM-G, DS, JH, EH-C, and LA analyzed and interpreted the data. RM-G, EH-C, and LA wrote the manuscript. All the authors revised the manuscript and approved its final version.

### Conflict of Interest

The authors declare that the research was conducted in the absence of any commercial or financial relationships that could be construed as a potential conflict of interest.

## Footnotes

^1^https://www.ncbi.nlm.nih.gov/genome/genomes/

^2^http://www.mbio.ncsu.edu/

^3^http://www.ebi.ac.uk/Tools/msa/clustalw2/

^4^https://embnet.vital-it.ch/software/BOX_form.html

^5^http://www.imtech.res.in/raghava/propred/

^6^http://imagej.nih.gov/ij
